# Metabolomics: A New Approach in the Evaluation of Effects in Human Beings and Wildlife Associated with Environmental Exposition to POPs

**DOI:** 10.3390/toxics10070380

**Published:** 2022-07-09

**Authors:** Miriam Acosta-Tlapalamatl, Claudia Romo-Gómez, Arely Anaya-Hernández, Libertad Juárez-Santacruz, Juan Carlos Gaytán-Oyarzún, Otilio Arturo Acevedo-Sandoval, Edelmira García-Nieto

**Affiliations:** 1 Ph.D. Program in Environmental Sciences, Autonomous University of State of Hidalgo, Mineral de la Reforma 42184, Hidalgo, Mexico; ac409820@uaeh.edu.mx; 2 Academic Area of Chemistry, Institute of Basic Science and Engineering, Autonomous University of State of Hidalgo, Carr. Pachuca-Tulancingo km. 4.5, Mineral de la Reforma 42184, Hidalgo, Mexico; claudiar@uaeh.edu.mx (C.R.-G.); acevedo@uaeh.edu.mx (O.A.A.-S.); 3 Research Center in Genetics and Environment, Autonomous University of Tlaxcala, Km 10.5 Autopista Tlaxcala-San Martín, Ixtacuixtla 90120, Tlaxcala, Mexico; arely.anayahernandez@uatx.mx (A.A.-H.); liber_05@yahoo.com.mx (L.J.-S.); 4 Academic Area of Biology, Institute of Basic Science and Engineering, Autonomous University of State of Hidalgo, Carr. Pachuca-Tulancingo km. 4.5, Mineral de la Reforma 42184, Hidalgo, Mexico; jcgaytan@uaeh.edu.mx

**Keywords:** metabolomics, biomarkers, POPs, disease, environmental exposure

## Abstract

Human beings and wild organisms are exposed daily to a broad range of environmental stressors. Among them are the persistent organic pollutants that can trigger adverse effects on these organisms due to their toxicity properties. There is evidence that metabolomics can be used to identify biomarkers of effect by altering the profiles of endogenous metabolites in biological fluids or tissues. This approach is relatively new and has been used in vitro studies mainly. Therefore, this review addresses those that have used metabolomics as a key tool to identify metabolites associated with environmental exposure to POPs in wildlife and human populations and that can be used as biomarkers of effect. The published results suggest that the metabolic pathways that produce energy, fatty acids, and amino acids are commonly affected by POPs. Furthermore, these pathways can be promoters of additional effects. In the future, metabolomics combined with other omics will improve understanding of the origin, development, and progression of the effects caused by environmental exposure.

## 1. Introduction

The presence of a wide range of compounds in the environment, coming from anthropogenic sources such as industrial processes, agricultural activities, combustion of wood and fossil fuels, incinerators, and uncontrolled landfills, has generated a negative impact on ecosystems, representing a risk for the wildlife and human health [1]. Compounds include the persistent organic pollutants (POPs), a variety of organic chemicals that feature a slow rate of biological, photolytic, and chemical degradation [2]. Due to these characteristics, the POPs can persist for an extended period in the environment, even at trace concentrations. POPs can be found in various environmental compartments, such as air [3], soil [4], food [5,6], sediments, and water [7]. Likewise, they can be transported long distances by wind and water currents, far from where they are used and released [8,9,10]. Their high lipid solubility gives rise to accumulation in fatty tissue and passes from one lower trophic level to the next through the food chain. They can enter the body through inhalation, ingestion, and dermal pathway [11].

The POPs are toxic chemicals of significant concern that adversely affect wildlife and human health worldwide. Endocrine disruption, reproductive, hepatic, neurological, and immune dysfunction, behavioral changes, and mutagenic and carcinogenic effects have been reported [12,13,14,15,16,17].

To address this global concern, diverse countries joined forces to sign and establish the Stockholm Convention, which came into force in 2004, and is a mechanism to protect human health and the environment by reducing or eliminating the production, use, and/or release of POPs. Currently, 30 pollutants are regulated by categories: A) subject to elimination of production and use, B) restriction of production and use c) reduction of unintentional release (Table 1) [18].

Once POPs enter the environment, wildlife and human beings are exposed to them. Consequently, various methodologies have been designed to assess or estimate the potential risk that the POPs pose to biota, which involves environmental analysis and mathematical modeling. The first encompasses measuring a broad range of analytes incorporated into environmental matrices using highly sensitive analytical instruments and techniques. The second methodology implies mathematical tools useful for simulating the physicochemical processes involved in the environmental kinetics and bioavailability of pollutants [19].

However, the risk estimation through the environmental assessment is not enough to guarantee the absence of adverse effects because both the individual compounds and the mixture or their possible transformations can modify their toxicity mechanism. From there, standardized toxicity assays can be employed to assess organisms’ responses; lethality and reproductive bioassays test to measure alterations in clinical signs and histopathological abnormalities [20]. Nevertheless, these techniques have limitations by ignoring the systemic effects produced by the pollutants. For this reason, new tools have been developed over the last two decades, such as omic biomarkers, which include the analysis of a set of molecular data, especially genomic, proteomic, and metabolomic biomarkers, to elucidate adverse effects and possible mechanisms of toxicity [21].

Omic biomarkers are promising tools for detecting subclinical effects associated with exposure to environmental pollutants and therefore play an essential role in risk assessment. However, in order for them to reach their maximum potential, their validation is required, through well-structured studies, analyzing and relating the exposure to a compound with the response of a biomarker or sets of them and deciphering that response as a transitory or specific event [22].

For all of the above, this review addresses the current state of studies conducted on the employment of metabolomics in assessing adverse effects on wildlife and human health due to environmental exposure to POPs. The information search covers the last ten years, using Web of Science and Pubmed, specialized search tools. These publications were selected by exploring the metabolomics topic and combining with the following words: wildlife, POPs, environmental exposure, mammals, fishes, poultry from wildlife, humans, organochlorinated pesticides, and each one of the 30 POPs individually. Studies carried out in vitro and/or involving the manipulation of organisms under controlled conditions were excluded.

## 2. Application of Omics in the POPs Assessment

Omics are a set of disciplines focused on obtaining a significant quantity of molecules involved in the functioning of an organism. Accordingly, these have been included in diverse fields of study to delve into and improve the collection of specific responses on the effects caused by environmental stressors [23].

Among these fields comes ecotoxicogenomics, defined as the integration of omics technology in ecotoxicology studies [24]. That is to say, it is the study of gene expression (Genomics), proteins (Proteomics), and the identification–quantification of endogenous and/or exogenous metabolites (Metabolomics) in wildlife and human population as the response in the light of exposure to environmental pollutants [25,26,27]. It is becoming a promising tool by increasing the sensitivity and specificity of other risk assessment criteria. It has elucidated the mechanisms of pollutants toxicity and helped us to understand how environmental toxicants are associated with responses at complex organizational levels such as populations and ecosystems, and has also contributed to the monitoring of adverse effects in organisms exposed to polluted environments [23,27,28].

The application of ecotoxicogenomics is still at a starting point. Most studies currently have been carried out on model organisms under controlled conditions. However, the challenge is to assess the risk to wildlife under natural conditions to better understand population dynamics [19].

### 2.1. Metabolomics

One of the tools employed for ecotoxicogenomics is metabolomics, which takes charge of a comprehensive analysis of endogenous and exogenous metabolites in cells, tissues and/or biofluids in response to diverse factors such as lifestyle, genetic effects, various pathologies, and environmental stressors [29].

The Human Metabolome Database (HMDB) currently lists around 250,000 total metabolites [30,31]. They perform multiple functions in the body, including signaling cascades, energy production, and macromolecule synthesis. Consequently, a metabolic alteration can unleash an adverse effect or exacerbate an existing one [29,32]. Such an alteration can correspond to the modification of a specific metabolite or the changes pattern of several metabolites. Therefore, their identification has become a new generation of biomarkers [33].

Furthermore, metabolomics has advantages over other omic technologies, such as processing a smaller number of biomolecules compared to the amount analyzed in genomics and proteomics. Likewise, metabolites have a well-conserved chemical structure in all organisms representing the final products of the cell regulatory processes. Therefore, the response of biological systems facing a variety of stressors is being best represented by it. In addition, the biological sample collection is less invasive, allowing multiple measurements to assess the temporal effects. On the other hand, the concentration of metabolites can change significantly, even though an enzyme concentration or metabolic fluxes does not alter [34,35].

The high sensitivity and efficacy of metabolomics in analyzing the metabolic pathways responses in cells, tissues, and biofluids exposed to environmental stressors promises to be important in the ecological risk assessment through the identification of new biomarkers and toxicity mechanism of pollutants [36].

### 2.2. Methodologies and Techniques in Metabolomics

In general, metabolomics studies follow a steps sequence to obtain the desired results: (1) study purpose, (2) sample collection and processing, (3) metabolite detection and quantification, and (4) data processing.

The first step is to determine the focus of the study to be conducted. For this, it is necessary to define whether analyzing as many metabolites as possible or only a specific group is required. Regarding the former, metabolomics has two approaches: untargeted and targeted focus [37]. The first one concerns obtaining data about the modifications of the greatest number of metabolites found in the sample, which allows for generating a hypothesis that gives way to more specific studies. The second approach is aimed to identify and quantify a finite number of metabolites according to the pre-established research hypothesis [38].

The second step is crucial in metabolomics analyses because it consists of collecting and processing the sample. However, acquiring and preserving samples under optimal conditions is essential to achieve adequate, reliable, and comparable results [39]. Blood plasma [40], urine [41], saliva [42], and amniotic fluid [43] are the biofluids most used by metabolomics, as well as different cells and tissues [44].

Depending on the biofluid or tissue, a specific treatment is carried out to extract the sample metabolites. In most cases, this consists of applying extraction techniques in a solid or liquid phase. Once the extracts have been obtained, they are stored at low temperatures until analysis [45].

Thirdly, metabolomics detects and quantifies metabolites using Nuclear Magnetic Resonance (NMR) and Mass Spectrometry (MS) techniques. NMR is commonly used in untargeted exploratory screening, and its advantages include its speed and high reproducibility by measuring multiple metabolites at once without requiring complex processing and sample destruction. The quantification of metabolites is carried out by comparing the areas of the spectral peaks with the internal reference standard. NMR provides partial information on the chemical structure of the molecule. As a disadvantage, NMR has low sensitivity and resolution compared to MS techniques [45].

On the other hand, MS requires sample processing through the use of separation techniques such as gas chromatography (GC) and high-performance liquid chromatography (HPLC) [46,47]. Usually, this process is complex because several chromatographic separations are often necessary (up to 72 h per sample), and specialized staff is required [48]. However, its sensitivity is high thanks to the extensive development of mass analyzers that allow both qualitative and quantitative metabolite profiles to be obtained. Major analyzers include single, triple, and time-of-flight (Q-TOF) quadrupole instruments, Ion Cycloton Resonance (ICR-FTMS), and Orbitrap, making it ideal for targeted analysis [49,50,51].

Using tandem mass spectrometry (MS/MS) is very useful for analyzing target compounds at trace levels (ppt-ppb range), and when high chemical noise is observed or the co-elution of characteristic ions. When the structure of the compound is unknown and/or additional structural information is required, MS/MS should be used. MS/MS exhibits higher sensitivity and specificity of the assay, especially in very complex matrices with the presence of interferences, such as fluid and tissue samples [52].

As the fourth step, data processing is accomplished, which turns out to be the most challenging since it consists of obtaining the raw data from the analytical techniques employed and converting them into data that allow the metabolites to be easily identified in data mining afterward. Once the raw data have been collected, these are analyzed through a database or spectral library searching; some used are Human Metabolome Database (HMDB), METLIN, MetaboLights, the Metabolomics Workbench, and Lipid Maps, KEGG, MassBank, SpectraBase, and BMRB) [30].

After this identification, the data sets are usually vast, so data mining tools are employed. For example, principal components analyses, partial least squares, discriminant analyses, and orthogonal projection to latent structures are used to identify significant differences, patterns, or correlations among data groups [53].

Finally, the pathways involved in the metabolic profile molecules are analyzed, thus identifying those that may be participating in a particular disease. For this, various databases contain pathways of multiple organisms, such as KEGG, Reactome, HumanCyc, SMPDB, HMDB, and MetaboAnalyst [54]. The latter is the most widely used (>300,000 users) as it allows high-throughput analysis in targeted and non-targeted metabolomics and integrates pathway topology and enrichment analysis for 26 model organisms with over 1600 pathways [55].

## 3. Description of the Population Evaluated with a Metabolomic Approach

From our own experience in the field, we identified 15 publications that applied metabolomics in populations environmentally exposed to POPs. Three corresponded to wildlife assessment (Table 2) and the remaining twelve to the human population (Table 3).

Atlantic bluefin tuna, *Thunnus thynnus*, is an essential species from the commercial and ecological standpoint within the Atlantic and Mediterranean ecosystems. However, its overexploitation as a fishery resource has placed it in the category of “endangered species”. In addition, being one of the main pelagic predators, it is prone to bioaccumulate and biomagnify environmental pollutants such as POPs that can induce adverse effects on its populations. Maisano et al. [56] evaluated the health state and metabolite changes between sexes by exposure to PCBs and organochlorine pesticides. They reported a 73% decrease in glucose and an increase in the level of malonate (178%), acetoacetate (80%), and acetone (19%) in males. Such metabolites are involved in fatty acid biosynthesis and ketogenesis, suggesting a possible onset of steatosis. On the other hand, Cappello et al. [57] found a significant increase in creatine, glucose, and glycerophosphocholine in females, as well as a decrease in choline, phosphocholine, amino acids (isoleucine, leucine, valine, alanine, sarcosine, and tyrosine), and energy-related metabolites (acetate, acetone, acetoacetate, malonate, lactate).

**Table 2:** Metabolomics studies evaluating the effects of environmental exposure to POPs in wildlife.

Analytic Method	POPs	POPs Concentration (ng/g Dry Weight)	Specie	Tissue or Biofluid	Associated Effect	Altered Metabolites	Reference
^1^ H-NMR	Ʃ DDT	M = 18.69 F = 24.31	Red tuna of the Atlantic *(Thunnus thynnus)* (*n* = 20)	Liver	Alteration in the energetic metabolism	Decrease of glucose; Increase of malonate, acetate, and acetone	[56]
	Ʃ 7 PCB-DL	M = 16.69 F = 7.94					
	Ʃ 6 PCB-NDL	M = 130.78 F = 53.27					
NMR	Ʃ 2 PFSA	264 ± 130	Polar bear (*Ursus maritimus)* (Females *n* = 112)	Plasma	Alteration in the metabolism of lipids	Glucose, lactate, HDL, triglycerides, cholesterol	[58]
	Ʃ 6 PFCA	81.7 ± 38.0					
^1^ H-NMR	Ʃ DDT	M = 18.69 F = 24.31	Red tuna of the Atlantic *(Thunnus thynnus)* (Males = 10) (Females = 10)	Liver	Alteration of the metabolic pathways producer of energy	14 aminoacids (isoleucine, leucine, valine, threonine, alanine, lysine, proline, sarcosine, taurine, glycine, tyrosine, phenylalanine, glutamate, and creatine; 9 metabolites of energy (acetate, acetone, acetoacetate, succinate, malonate, malate, lactate, glucosa, fumarate); 1 nucleoside (Inosine) 9 diverse metabolites (isopropanol, glutathione, choline, phosphocholine, niacinamide, hypoxanthine, glycerophosphocholine, and glycerol)	[57]
	Ʃ 7 PCB-DL	M = 16.69 F = 7.94					
	Ʃ 6 PCB-NDL	M = 130.78 F = 53.27					

Persistent Organic Pollutants (POPs), male (M); female (F), Ʃ DDT (2,4′DDE; 4,4′DDE; 2,4′DDD; 4,4′DDD), Ʃ 7 PCB-DL (PCB 105, 118, 123, 126, 156, 157, 167), Ʃ 6 PCB-NDL (PCB 28, 52, 101, 138, 153, 180), Σ 2 PFSA (perfluoroalkyl sulfonates with 6 and 8 carbons), Ʃ 6 PFCA (perfluoroalkyl carboxylates with carbon chain length of 8 to 13). ^1^: Proton Nuclear Magnetic Resonance (1).

**Table 3:** Studies that employ metabolomics to evaluate the effects associated with the exposure to POPs in human population.

Analytic Method	POPs	Concentration	Population/Exposure Type	Tissue/Biofluid	Effect Associated	Altered Metabolites	Reference
UHPLC-QTOF-MS	Dioxin	(~ 5000 pg/g lipid)	11 workers from a herbicide production plant. Occupational	Urine	Alteration of endogenous steroid metabolites and profiles of urinary, biliary acids	Glucuro and sulfoconjugates of glycochenodeoxycholic acid, estrone glucuronide, glycocholic acid-3-glucuronide, glycoursodeoxycholic acid glucuronide and sulfate, hydroxytestosterone glucuronide, hydroxyandrosterone glucuronide, Dihydrotestosterone sulfate, glucuro and sulfoconjugates of androsterone, Dihydroxyandrostenone sulfate, Isomer of epitestosterone glucuronide, glycocholic acid, chenodeoxycholic acid sulfate, hydroxyandrostane glucuronide, pregnanediol-3-glucuronide, cholic acid glucuronide, deoxycholic acid glucoronide	[59]
UPLC-QTOF-MS	p,p′-DDE	309 ng/g lipid	965 older men and women Environmental	Plasma	Changes in lipid metabolic pathways include fatty acids, Glycerophospholipids, Sphingolipids and glycerolipids	Oleic acid amide, heptadecanoic acid, linolenic aldehyde, flavone, Lysophosphatidylcholine (18:1), Lysophosphatidylcholine (0:0/18:2), Lysophosphatidylcholine (18:2/0:0), Lysophosphatidylcholine (18:3), Monoacylglycerol (18:2), Phosphoethanolamine ceramide (34:1), Phosphoethanolamine ceramide (36:1),cinnamic acid and its derivatives, docosahexaenoic acid, lysophosphatidylethanolamine (18: 1p/0: 0), Lysophosphatidylethanolamine (18: 1b), Lysophosphatidylethanolamine (18:2)	[60]
	HCB	40.8 ng/g lípid					
^1^ H-NMR	Β-HCH	21.4–46.8 ng/g lípid	750 Pregnant women from general population Environmental	Plasma	Changes in: Mitochondrial catabolic pathway of the L-leucine and in the metabolism f organic acids	3-hydroxyisovalerate (decrease), 4 deoxyerythronic acid, succinate, Pregnanolone-3G, Alanine, Glycine, 3-hydroxybutyrate/3-Aminoisobutyrate, acetone.	[61]
	HCB	21.6–66.6					
	DDE	75.5–201					
	PCB138	11.6–27.7					
	PCB180	15.9–34.3					
	PCB180	15.9–34.3					
	PFOAS	1.69–3.67					
	PFOS	3.94–8.15					
	PFNA	0.557–1.05					
	PFHxS	0.686–1.14					
ICR-FTMS	PFOA	1.88–5.37 ng/mL	19 boys and 21 girls Hispanic Environmental	Plasma	Deregulation of metabolic pathways of lipids, amino acids, and glucose	Glycosphingolipids, fatty acids, linoleic acid, asparagine, tyrosine, arginine and proline	[62]
	PFOS	1.95–65.3 ng/mL					
	PFHxS	0.47–12.81 ng/mL					
UHPLC-FTMS	PFOAS	2.6 ng/mL	49 boys and 66 girls from Cincinnati Environmental	Plasma	Alteration of the metabolism of amino acids and lipids	Arginine, proline, aspartate, asparagine, beta-alanine, butanoate, glutamate, glycerophospholipids, glycine, serine, alanine, threonine, glycosphingolipids, Gloxylate, Dicarboxylate, histidine, Linoleate, methionine cysteine, tyrosine, urea, Tianima and nicotinamide.	[63]
	PFOS	4.4 ng/mL					
	PFNA	0.9 ng/mL					
	PFHxS	2.1 ng/mL					
UHPLC-Orbitrap-MS	PBB-153	5.3–53.2 ng/g	68 men and 88 women from Michigan Environmental	Plasma	Changes in the metabolic pathways of the catecholamines, the cellular respiration, the essential fatty acids, the lipids, and polyamines.	Asparagine, Threonine, Retinyl beta-glucuronide 25-hydroxyvitamin D2, 1 alfa, 24R, 25-trihydroxyvitamin D3, Leukotriene B4,Sphinganine, Creatine, Acetylcarnitine, Succinate, Citrate;Iso-cittrate Glucose, Cytosine, 5-hydroxy-N-formylquinurenine, Dopamine, Putrescine, N-acetyl-L-glutamate 5-semialdehyde, Picolinic acid, 5,10-methylenetetrahydrofolate, Prostaglandin B1 N-acetyl-L-glutamate 5-phosphate, Uridine triphosphate 3-(4-hydroxyphenyl) pyruvate, 3,4-dihydroxy-L-phenylalanine 3-methoxytyramine, Glycine, Selenohomocysteine Tryptophan, Pyridoxamine, Retinyl beta-glucuronide, Linoleic acid, Glycolate, Dihydrobiopterin, Tetrahydrobiopterin, Spermine Dialdehyde N-methylputrescine, N8-acetylspermidine, Cortisol, serine, Eicosadienoic acid Phosphoethanolamine, Cer (d18: 0/22: 0) PI (16: 0/20: 0), Palmitoylcarnitine, Uracil, Urocortisol	[64]
	PCB-153	9.9–20.5 ng/g					
UHPLC-Orbitrap-MS	PFAS	1.61–3.18 ug/L	58 men and 44 women with obesity or over- weight Environmental	Plasma	Alteration of the metabolic pathways of fatty acids, lipids, and amino acids.	Arginine, proline, tryptophan, hexoses	[65]
	PFOS	1.61–11.47 ug/L					
	PFHxS	0.32–5.79 ug/L					
UHPLC-Orbitrap-MS	p,p′-DDE	42.81 ng/mL	50 women with breast cancer Perinatal	Maternal perinatal serum	Alteration of the metabolic pathways of amino acids, glycerophospholipids, fatty acids, and the cycle of urea	Pipecolate, semialdehyde, Hydroxyglutamate, Methylphenylethanolamine, Arginine, sarcosine, tyramine, 4-acetamidobutanoate, 2-Amino-3-oxobutanoic acid, Betaine, (-)—Salsolinol, 2-phenylacetamide, 4, Fumarylacetoacetate, Indol-5, 6-quinone	[66]
UHPLC-Orbitrap-MS	PFOA	3.42 ng/mL	52 boys and 22 girls with NAFLD	Liver	Changes in the key pathways of amino acids and lipids underlying the pathophysiology of the NAFLD	Increase of: Phosphoethanolamine, Tyrosine, phenylalanine, Aspartate and creatine Decrease of: Betaine	[67]
	PFOS	3.59 ng/mL					
	PFHxS	1.53 ng/mL					
UHPLC-Orbitrap-MS	17 dioxin congeners	(3.29–765.35 pgTEQ/g lipid)	95 Workers from a waste incineration power plant and two electronics factories Environmental	Plasma	Changes in the metabolism f the β-oxidation of the fatty acids, Glycerophospholipids, sphingolipids, essential fatty acids, purines, aminoacids	Tetradecanoylcarnitine, Decanoylcarnitine, L-palmitoylcarnitine, Palmitamide, 3-hydroxy caproic acid, Prostaglandin H2 (PGH2), Arachidonic acid (AA), Stearidonic acid, 9-OxoODE, Octadecanamide, Glycerophospho-N-palmitoyl ethanolamine (GP-NPEA), N-Oleoylserine, PC (18:1/18:1), LPC (16:0/0:0), LPE(16:0/0:0), Sphingosine-1-phosphate (S1P), Adenosine monophosphate (AMP), Xanthine, Indolactic acid and aspartic acid.	[68]
UHPLC-QTRAP-MS	*Trans*-nonachlor	3.88–9.59	26 women without endometrioma; 49 women with endometrioma Environmental	Plasma	Dysregulation of bile acid homeostasis and lipase activity: Higher concentrations of POPs are associated with a higher risk of endometrioma	Interleukin-8, monocyte chemoattractant protein-1, triglycerides, lysophosphatidylcholines, phosphatidylcholines, ceramides, fatty acids	[69]
	PCB-114	128.17–255.70					

^1^: Proton Nuclear Magnetic Resonance (1).

Knowing how POPs induce adverse effects on the energy metabolism of Arctic wildlife species is crucial to understanding how they will respond to changes in their habitat. Tartu et al. [58] reported that POPs coupled with declining sea ice exert a synergistic adverse effect on lipid biosynthesis and catabolism in female polar bears.

The studies have dealt with diverse population groups, ranging from 7 to 75 years regarding the human population. Therefore, risk groups such as children [62,63,65,67], pregnant women [61], and elderly adults [60,70] have been considered. Most of the studies used plasma as the main biofluid to perform the metabolomic profiles, and the most applied analytic method was liquid chromatography coupled with mass spectrometry. It is worth mentioning that studies with this approach have increased in recent years, from one study in 2014 to five in 2020. The United States is the top country where these studies have been carried out.

Epidemiological studies with a metabolomic approach have enabled an understanding of how some POPs induce metabolism alterations and are associated with some pathologies. For example, Alderete et al. [62] reported an increase in glucose and a significant alteration of the lipids and aminoacid pathways in Hispanic adolescents with obesity or overweight; these alterations could be an underlying key to type 2 diabetes. In this study, 97.5% of participants showed high perfluoroalkyl substances (PFAS) concentrations. In another study involving young adults, 49% of whom were overweight, alterations in the metabolic pathways of lipid and amino acids were associated with a greater risk of cardiometabolic disease [65].

On the other hand, Jin et al. [67] carried out the first study about PFAS exposure and its association with the seriousness of Non-Alcoholic Fatty Liver Disease (NAFLD) in children. They reported an increased risk that children diagnosed with NAFLD would develop steatosis due to the high plasmatic concentrations of PFAS and the changes produced in the metabolic pathways of amino acids and lipids. In another group of children, serum PFAS concentrations were associated with alterations in the Tricarboxylic Acid cycle related to energy-producer pathways and catabolism [63].

Pregnancy is another crucial stage where exposure to environmental pollutants can have adverse consequences on both the mother and fetal development and subsequent infant health. Therefore, it is essential to identify metabolic signatures associated with such exposure, as in the study reported by Maitre et al. [61], where the metabolic profiles in urine samples at 12 and 32 weeks of pregnancy from women exposed to PCBs showed a decrease in 3-hydroxyisovalerate, a product of the mitochondrial catabolic pathway of L-leucine. For the case of PFAS and organochlorine pesticides, no consistent associations were found. On the other hand, Hu et al. [66] analyzed maternal serum samples during pregnancy and early postpartum, reporting an association between the concentrations of p,p′-DDT, o,p′-DDT, and p,p′-DDE with alterations in the metabolic pathways of glycine, serine, alanine, threonine, urea cycle, catabolism of non-essential amino acids, glycerophospholipids, fatty acids, carnitine, and glucose.

It was also reported in a cohort of Swedish older adults that serum levels of organochlorine pesticides such as p,p′-DDE, and HCB are associated with variation in lipid metabolism, including fatty acids, glycerophospholipids, sphingolipids, and glycerolipids. Moreover, 16 metabolites associated with the exposure were identified, including lipids related to cell signaling, energy regulation, and membrane composition [60].

In another study, a group of individuals participating in the Michigan PBB registry exposed to PCB-153 and PBB-153 was evaluated. Metabolites that were associated with PCB-153 included 4-hydroxyphenylpyruvic acid (4-HPAA), 3,4-dihydroxy-L-phenylalanine (L-dopa), and 3-methoxytyramine (3-MTT). These partake in the production of dopamine and, even though these were not associated with PCB-153, there was a positive correlation with the PBB-153. It indicates that both compounds interact in catecholamines metabolism and are associated with alterations in metabolic pathways of cellular respiration, essential fatty acids, lipids, and polyamines. These were consistent with pathophysiological changes observed in neurodegenerative diseases like Parkinson’s [64].

Endometriosis are other diseases associated with environmental exposure to POPs and have been evaluated. Using targeted metabolomics tools, Matta et al. [67] associated the presence of *trans*-nonachlor and PCB 114 with an increased risk of endometriosis in French women. Additionally, they found a high inflammatory profile (interleukin-8 and monocyte chemoattractant protein-1) and alterations in bile acids and lipase activity.

Similarly, high levels of dioxins in serum have been associated with a potential risk of developing cardiovascular and liver disease due to the alteration of metabolic pathways, such as the β-oxidation of fatty acids, the metabolism of essential fatty acids, arachidonic acid, glycerophospholipids, sphingolipids and purines [68].

Urine is one of the most challenging biofluids because of the significant number of confusing factors that it presents. Hence the application of studies with a targeted approach allows better control of any biases that may arise. Jeanneret et al. [59] reported changes in urine as an outcome of dioxin exposure in a group of Czech workers. Results showed altered endogenous steroid metabolites levels and urinary bile acid profiles, consistent with increased cytochrome P450 expression, persistent hepatotoxicity, dysregulation of bile acid homeostasis, and oxidative stress.

## 4. Challenges and Perspectives

Technological advances that employ metabolomics have allowed wide access to the information contained in the human metabolome. Nevertheless, it presents several challenges, such as properly interpreting most data gathered to improve and maximize knowledge about the objectives stated [71].

Furthermore, the lack of reference standards for many of the metabolites impedes their identification as it is not possible to characterize the metabolite from the fragments that compose it, since a large part of the metabolites are common in all species, and for this reason, fragmentation patterns can be unpredictable or provide little information [72,73]. 

Until now, there is a lack of a universal analytical method capable of identifying and quantifying the entirety of metabolites present in biological samples, mainly due to the instability of the metabolites under analytical conditions. It could cause considerable degradation of the target metabolites and also produce analytical bias, coupled with the presence of a large amount of chemical substances that are not of interest but can be detected by mass spectrometry [74,75].

The costs involved in performing a metabolomic analysis could be relatively low compared with the genomic or proteomic biomarkers. However, the investment required in analytical instrumentation remains high, making it difficult for many laboratories to access this technology. Moreover, human resources highly trained in diverse areas are required, and it is not possible to have a multidisciplinary group in all laboratories [76].

Among the perspectives towards the future, the possibility that metabolomics begins to commercialize laboratory tests is not ruled out, provided that there is a significant demand of users and therefore profitable. On the other hand, once the specific metabolites that participate in certain pathologies are correctly identified, portable tools of easy access could be developed. These could provide fast and reliable results easily interpretable by users [77,78].

It is also worth mentioning that one of the great perspectives of metabolomics is its combination with other omic technologies, which allows an assessment at each of the different levels of molecular organization to be carried out and elucidates the bioprocesses that control the metabolome in the same study [79]. Related to this latter, a better understanding of the role of a metabolite identified as a biomarker of some specific pathology could be obtained; this is due to the fact that metabolites originate in multiple metabolic pathways. Therefore, although the use of metabolomics as the single analysis tool can detect disease biomarkers, it provides little or no information about the processes that give rise to a certain alteration [80,81].

In this way, systems biology tries to integrate metabolomics and all the other omics to carry out a holistic analysis of organisms that conduces to a better understanding of living systems and enables the possible prediction of their behavior. Furthermore, it allows for addressing health-disease as a whole and not as the analyses of its parts separately, understanding that vital functions and their pathological manifestations, as well as the possible treatments, are the results of complex interrelationships among multiple levels of molecular organization [82,83].

## 5. Conclusions

The health status of the human population and wildlife is compromised by exposure to a complex mixture of environmental stressors. Hence it is crucial to understand how such stressors exert their toxic effect. For this purpose, metabolomics has become a powerful tool for identifying various metabolites that function as early effect biomarkers associated with diverse pathologies; this enables a better diagnosis, prevention, and/or proper treatment. Notwithstanding, it is necessary to continue with this type of investigation, expanding the number of species and individuals participating in each study; this enables comparing and extrapolating the results obtained.

Even though metabolomics faces challenges, the rapid evolution in the field of omics and bioinformatics technologies helps to counteract them and project these as highly useful tools in multiple areas, such as precision medicine and environmental risk assessment.

Studies employing metabolomics in the human population and wildlife environmentally exposed to POPs have increased in the last years, indicating that this omics approach is in an early stage with a long way to go in exploration and discovery. Therefore, soon this tool can be combined with other omics to obtain a holistic understanding of the origin, development, and progression of pathology.

## Figures and Tables

**Table 1:** POPs considered in the Stockholm Convention.

	Classification					
POPs	A	B	C	Pesticides	Industrial Chemicals	Unintentional Production
Perfluorooctanoic acid (PFOA), its salts and related compounds with PFOA	x				x	
Perfluorooctane sulfonic acid, its salts and Perfluorooctane sulfonyl fluoride		x		x	x	
Aldrin	x			x		
Polychlorinated biphenyls (PCB)	x		x		x	x
Chlordane	x			x		
Chlordecone	x			x		
Dichlorodiphenyltrichloroethane (DDT)		x		x		
Decabromodiphenyl ether (commercial mixture, c-decaBDE)	x				x	
Polychlorinated dibenzofurans (PCDF)			x			x
Polychlorinated dibenzo-p-dioxins (PCDD)			x			x
Dicofol	x			x		
Dieldrín	x			x		
Technical endosulfan and its related isomers	x			x		
Endrin	x			x		
Heptachlor	x			x		
Hexabromobiphenyl	x				x	
Hexabromocyclododecane (HBCDD)	x				x	
Hexabromodiphenyl ether and heptabromodiphenyl ether	x				x	
Hexachlorobenzene (HCB)	x		x	x	x	x
Hexachlorabutadiene (HCBD)	x		x		x	x
Lindane	x			x		
Mirex	x			x		
Polychlorinated naphthalenes	x		x		x	x
Short-chains chlorinated paraffin (PCCC)	x		x		x	x
Pentachlorobenzene	x		x	x	x	x
Pentachlorophenol and its salts and esters	x			x		
Tetrabromodiphenyl ether and pentabromodiphenyl ether	x				x	
Toxaphene	x			x		
α-hexachlorocyclohexane	x			x		
β- hexachlorocyclohexane	x			x		

## Data Availability

Not applicable.
